# Satisfaction with Hearing Aids Based on Technology and Style among Hearing Impaired Persons

**Published:** 2016-09

**Authors:** Farzad Faraji- Khiavi, Rezvan Dashti, Seyyed-Jalal Sameni, Arash Bayat

**Affiliations:** 1 *Department of Health Services Administration, School of Health, Ahvaz Jundishapur University of Medical Sciences, Ahvaz, Iran.*; 2 *Department of Rehabilitation Administration, Musculoskeletal Rehabilitation Centre, School of Rehabilitation, Ahvaz Jundishapur University of Medical Sciences, Ahvaz, Iran.*; 3 *Department of Audiology, School of Rehabilitation. IUMS Lecturer, Iran Medical Sciences University, Tehran, Iran.*; 4 *Hearing and Speech Research Center, School of Rehabilitation, Ahvaz Jundishapur University of Medical Sciences, Ahvaz, Iran. *

**Keywords:** Hearing aid, Hearing loss, Hearing impaired persons, Satisfaction

## Abstract

**Introduction:**

Hearing loss is one of the most disabling impairments. Using a hearing aid as an attempt to improve the hearing problem can positively affect the quality of life for these people. This research was aimed to assess satisfaction of hearing impaired patients with their hearing aids regarding the employed technology and style.

**Materials and Methods:**

This descriptive-analytic cross-sectional research was conducted on 187 subjects with hearing loss who were using a hearing aid. The subjects were over 18 years of age and were using a hearing aid for at least 6 months. The Persian version of Satisfaction with Amplification in Daily Life (SADL) questionnaire was the instrument which was used for assessing satisfaction with the hearing aid. Cronbach’s alpha was calculated to be 0.80 for instrument reliability.

**Results:**

A significant difference was observed among satisfaction subscales’ mean scores with hearing aid technology. Also a significant difference was observed between the total satisfaction score and the hearing aid model. With respect to the analysis of satisfaction with the hearing aid and its style, cost and services was the only subscale which showed a significant difference (P=0.005).

**Conclusion:**

Respondents using hearing aids with different technology and style were estimated to be quite satisfied. Training audiologists in using more appropriate and fitting hearing aids in addition to using self-reporting questionnaires like SADL for estimating patients’ social condition and participation in their life can essentially change their disability condition and countervail their hearing loss.

## Introduction

WHO (2013) estimated that 360 million people in the world are suffering from disabling hearing loss (1). In recent years, hearing loss has not just been evaluated from a biological approach. Rather, it has also been considered from the economic, social, and personal approaches when involvement in communication with others and participation in community are concerned (2). Hearing loss can consequently lead to social isolation, less activity, and decreased quality of life (3). Hearing aids are the first practical step in aural rehabilitation process for the majority of those who suffer from hearing loss (1,4).

Under 25% of individuals who can improve by hearing aids are real users and this rate is lower in developing countries (5). It can be due to some factors such as getting labelled for using hearing aids, consumers’ dissatisfaction, and high costs of hearing aid and rehabilitation services (6).

The aim of using a hearing aid is amplifying signals that make sounds audible for hearing-impaired people. Basically, all hearing aids use analogue technology to amplify sounds (6). Although each hearing aid contains a microphone and a receiver system, their main difference is their function. Analogue hearing aids include some limited controls. Programmable hearing aids use digital control circuits and usually make a more accurate fitting than analogue hearing aids. In digital hearing aids, analogue input signals are converted to digital input and then the processes continue (7).

Advancements in digital technology and the rising speed of speech signal processing have ensued current developments in existing features of modern hearing aids. However, hearing aid users still have complaints hearing speech signals in noisy environments and while talking on the phone (8).

Digital hearing aids are more flexible for fitting and include more complex processing (7). They also have some extra features such as being multi-programmable and having automatic feedback control in comparison with customary hearing aids (9). From another perspective, digital hearing aids are getting smaller in size and consuming less power compared with the analogue ones (7). However, success in the hearing aid adaptation process depends on the user’s satisfaction with hearing aid results (10). Consumer satisfaction assessment is a key part of comprehensive assessment programs in health care (11). Satisfaction is a subjective phenomenon that shows patients’ concept of structures, processes, and the outcomes of delivered services (12). Studying the efficacy of rehabilitation services and the satisfaction with hearing aids in hearing-impaired people can result in delivering more appropriate services which are adjusted to their needs (2). Since desirable sound amplification influences the efficacy of aural rehabilitation, self-report questionnaires can be considered as appropriate instruments for assessing consequences of using hearing aids and the users’ satisfaction (12). Satisfaction of amplification in daily life (SADL) questionnaire is a self-reported questionnaire, developed by Cox and Alexander (1999), to evaluate user’s satisfaction in various dimensions of using such a device. This research aimed to assess satisfaction with hearing aids based on technology and style among hearing impaired people.

## Materials and Methods

This research was an analytical and cross-sectional study. The participants were hearing-impaired individuals who were referred to an audiology clinic in the south of Bushehr province, Iran. The inclusion criteria for participation in the study were: referring to a clinic in the last two years, over 18 years of age ,and at least a 6 months’ experience in using hearing aids. There was no exclusion criteria to participate in this research. The population size included 187 people all of whom consented with the research procedures. The population under study included 100 male and 87 female participants aged between 18 to 90. Initially, audiology evaluations were performed for the subjects. Then, they filled the questionnaire (for illiterate individuals, a reviewer read questions and marked their answers on the questionnaire). Subject evaluations included two phases: 

*1. Audiological Evaluation Including:*

Otoscopy for examining ear appearance and pure tone audiometry was performed by a calibrated audiometer in an acoustic room. In this evaluation audio absolute thresholds for Air Conditions (in octave and half-octave frequencies of 250-8000 Hz) and Bone Condition (in octave and half-octave frequencies of 250-4000 Hz) were determined. Speech audiometry evaluated Speech Recognition Thresholds (SRT) and word Recognition Score (WRS). Immitance audiometry was used for the evaluation of the accurate function of the sound transmission system to the inner ear.

*2. Satisfaction Assessment Instrument:*

A standard Persian version of SADL and a questionnaire for demographic characteristics were used for data gathering. The questionnaire was validated through face and content validity by 5 experts. Cronbach’s alpha calculated 0.80 for reliability of the data gathering instrument (13). The SADL questionnaire was developed for assessing satisfaction of hearing-impaired people with their current hearing aid. The questionnaire contained 15 questions and four subscales comprised of 1) Positive effects 2) Negative features 3) Services and costs, and 4) Personal image. Positive effects subscale included 6 questions about acoustic and psychological advantages of the hearing aid. Negative features encompassed three questions about amplifying background noise and acoustics as well as using a phone. Three questions about the skills of the prescribing specialist, hearing aid price, and repairing times were included in the cost and services subscale. Personal image was assessed in the last subscale involving three questions about motivation, cosmetic, and labelling factors with using the hearing aid. The mean score of these four subscales was used to assess a respondent’s satisfaction and was called his or her global score. A Likert seven-option scale was used for ranking the answers whose range varied from “strongly disagree” to “strongly agree” options. In 11 questions, choosing “strongly agree” meant complete satisfaction and scored 7, while choosing “strongly disagree” meant complete dissatisfaction and scored 1. Four questions were scored reversely and choosing “strongly disagree” meant complete satisfaction and scored 7. The questionnaire’s validity was approved by the developer in 2001. They declared instrument reliability was more than 0.83 for all of the questions. Demographic characteristics included age, sex, education, experience with hearing aids, and the daily use of hearing aids.

This project was conducted under the ethics committee of Ahvaz Jundishapur University of Medical Sciences (Ethics Approval No. ajums.rec.1393.5). All respondents declared and signed their consents formally. The data was analyzed by descriptive statistics (mean and standard deviation) and inferential statistical (independent T, ANOVA and LSD Post Hoc tests) in SPSS.

## Results

One hundred males and 87 females (range: 18 to 90 years old) were evaluated in this study. Demographic characteristics of the participants are presented in Table 1.

**Table 1:** Demographic characteristics of respondents

**Variable**		
**Age group**		
18-30	67	35.80
30-50	41	21.90
50-65	41	21.90
65 to up	38	20.40
**Sex**		
Male	100	53.50
Female	87	46.50
**Education**		
Illiterate	89	47.60
Not finished high school	74	39.57
High school Diploma and up	24	12.83
**Degree of Hearing loss (without hearing aid)**		
Moderate	87	46.50
Moderate to severe	71	38.00
Severe	29	15.50
**Daily hearing aid use**		
1-4 hours	12	6.40
4-8 hours	27	14.40
8-16 hours	148	79.20
**Experience with current hearing aid**		
6 weeks to 11 months	25	13.30
1 to 10 years	152	81.40
Over 10 years	10	5.30
Total	100	187

As seen in Table 1, the majority of subjects were illiterate. Most of them were recognized with moderate hearing loss. 

The majority of the participants (79.20%) used a hearing aid 8-16 hours per day. 14.40% and 6.40% of subjects were using their hearing aid respectively as long as 4-8 and 1-4 hours daily. Most respondents had been using their current hearing aid for 1 to 10 years. 

107 (57.21%), 22 (11.76%), and 58 (31.03%) subjects were using digital, programmable, and analogue hearing aids respectively. Satisfaction assessment results with hearing aid based on technology is shown in Table 2.

**Table 2:** Satisfaction with hearing aids and their types of technology

**Hearing Aid Technology**				
SADL subscale	Digital	Analogue	Programmable	P-value
Cost and Services	5.57±1.24**	4.33±1.22	4.80±1.14	>0.001
Personal Image	4.14±1.07**	4.75±0.87	4.75±1.05	>0.001
Negative Features	3.64±0.87*	3.41±0.73	3.24±0.72	0.027
Positive Effect	6.18±0.96	5.58±1.07*	6.18±0.82	0.078
Global score	5.14±0.55	4.84±0.67*	5.03±0.51	0.010

* Statistically significant at level 0.05

ANOVA test showed a significant difference in SADL subscales for different technologies of hearing aids. In the cost and services subscale, significant differences were seen between participants who used a digital hearing aid and the other two groups. In the personal image subscale, significant differences were observed between subjects with a digital hearing aid and those with analogue and programmable hearing aids (using LSD post hoc test).

In the global score, a significant difference was observed between people who used digital hearing aids and those with analogue hearing aids. Satisfaction level with hearing aids with different technologies was estimated at the same level. A maximum level of satisfaction was in the positive effect subscale where a high degree of respondents’ satisfaction was observed. A minimum level of satisfaction was observed in the negative features. Users were estimated relatively satisfied in the other two subscales.

Fifty (26.73%) respondents were using ITE types of hearing aids and 137 (73.27%) were using BTE ones. Results of assessing the satisfaction level based on the model of hearing aids are shown in Table 3. We found a significant difference between different hearing aid models in the global score (Table.3). Subjects with ITE hearing aids were significantly more satisfied in all subscales except for the “negative features”. Maximum level of satisfaction was seen in the positive effect subscale. 

**Table 3:** Satisfaction with hearing aids based on their models

**SADL subscale**	**Hearing Aid Model**			**P-value**
	ITE types		BTE types	
Cost and Services**	5.58±1.15	4.82±1.30		>0.001
Personal Image**	3.89±1.19	4.59±0.92		>0.001
Negative Features	3.51±0.83	3.53±0.82		0.83
Positive Effect**	6.50±0.66	5.93±1.04		>0.001
Global score**	5.25±0.50	4.96±0.61		0.002

** Statistically significant at level 0.05

Nineteen (10.16%) subjects were using a hearing aid binaurally and 168 (89.84%) were using it monaurally. Results of assessing the satisfaction level with a hearing aid based on the style of hearing aids are shown in Table 4. 

Our analysis demonstrated that users with binaural style of hearing aids were significantly more satisfied in the Cost and Services subscale. Other subscales showed no significant difference.

**Table 4:** Satisfaction with hearing aids and their styles

**SADL subscale**	**Hearing Aid Model**			**P-value**	
	Monaural		Binaural		
Cost and Services**	5.02±1.34	5.92±1.08		0.005	
Personal Image**	4.43±1.03	4.22±1.21		0.39	
Negative Features	3.52±0.81	3.60±0.89		0.69	
Positive Effect**	6.06±0.98	6.34±1.01		0.24	

** Statistically significant at level 0.05

173 (92.51%) subjects were suffering from sensorineural hearing loss and 14 (7.49%) were suffering from mixed hearing loss. No significant difference was observed in the satisfaction level of respondents with different kinds of hearing loss. In addition, satisfaction level showed no significant difference between the two genders.

**Fig 1:**
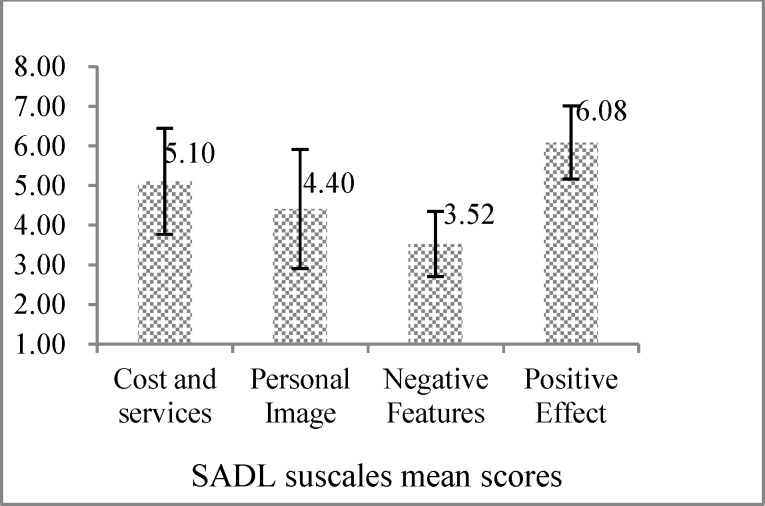
Mean scores distribution of global satisfaction with hearing aid and its dimensions

Figure 1 shows that the maximum level of satisfaction with hearing aids was in the positive effect subscale and the minimum satisfaction level was observed in the negative features.

## Discussion

Subjects were estimated to be relatively highly satisfied based on their mean global score in this study. This result is in agreement with the results of the studies by Cox and Alexander, Viega et al , and Carvalho (14-16).

In the present work, no significant difference was seen in satisfaction level with hearing aid between the respondents’ sex and age, which is similar to Uriarte et al's studies (12). In Hosphord-Dunn and Halpern, and Jerram and Purdy's studies, however, there was a significant difference between male and female participants (17,18). 

The result of the present study showed less satisfaction in age groups in comparison with Kochkin's research (19). Although Cox and Alexander did not report any correlation between satisfaction and age (14), it must be considered that their study population comprised respondents over 60 years old. Jerram and Purdy studied patients between 30 to 88 years of age (18), and Uriarte et al's study was conducted on age groups between 29 to 104 years of age (12). However, this study was done on subjects between 18 to 90 years of age and this difference of age groups in the samples of the study may explain why a similar result was not observed as compared with other investigations on the relationship between the participants' age and the satisfaction level of hearing aids.

Significant differences were seen between all subscales of satisfaction considering different technologies: people with digital hearing aids were estimated significantly more satisfied in cost and services, personal image, and negative features subscales. Also, patients with analogue hearing aids were estimated significantly less satisfied in positive effects subscale and global satisfaction. Yet, all respondents were estimated to be satisfied with their hearing aids, which is similar to Vuchrialho et al and Uriarte et al's findings (12,20). They explained technical development in hearing aid technology could cause more satisfaction and reported that the percentage of real users of hearing aids was higher than the previous 20 years. In this study, subjects were using one of the three kinds of technology in hearing aidz: digital, programmable, and analogue; while all of the subjects in Cox and Alexander’s study were using analogue hearing aids, so their results cannot be compared with this study. According to Kochkin, a hearing aid's programmability was accompanied by more satisfaction (19). This can explain differences in high scores of satisfaction in the present research compared with the results of other studies such as those of Cox and Alexander, Arlinger, and Kaplan- Neeman et al (14,21,22). Finally, despite all excessive advances in hearing aid designing as well as quality improvements, it seems that some factors such as the users’ dissatisfaction arising from disregarding their very high expectations in addition to the high cost of modern hearing aid leads to less use of hearing aids.

Moreover, a significant difference was observed between different models of hearing aids in the global score as well as the positive effect, the cost and services, and the personal image subscales in this study. Dillon et al and Kochkin reported a correlation between high satisfaction in the personal image subscale with ITE hearing aids, which supports our findings (19,23). In this research, no difference was observed between global satisfaction with hearing aids in monaural or binaural hearing aid styles. This is analogous with the results of Uriarte et al and Kochkin; however, higher satisfaction of participants with the binaural style of hearing aid was reported (12,19).

The positive effect subscale showed the highest mean among other subscales indicating the high satisfaction of hearing-impaired people with hearing aids in their social life. Considering the hearing aid's sound quality, only few subjects reported dissatisfaction with acoustical specifications and psychological effects of their hearing aid. This result confirms the outcomes of Cox and Alexander as the developers of the study's questionnaire (14).

## Conclusion

In this study, satisfaction level with digital hearing aids was estimated to be higher than other types of hearing aids. However, these types of hearing aids impose higher costs on the users. Establishing policies in order to remove access financial barriers to these types of hearing aids need to be studied. Since the prevalence of hearing loss as well as the need for its rehabilitation is growing, these rehabilitation services need to be supported by social security and retirement funds. These organizations must specify which groups are in priority for using these resources and which patients gain more advantage with hearing aids. 

Given the lower satisfaction level with their hearing aids among illiterate subjects in this study, more counselling meetings for these patients and spending more time for instructing these customers in using their hearing aid is recommended. Besides, providing an educational protocol for using amplification in daily life can lead to better results.
